# Hydrogen Evolution Reaction Performance of Ni–Co-Coated Graphene-Based 3D Printed Electrodes

**DOI:** 10.1021/acsomega.2c07856

**Published:** 2023-02-02

**Authors:** Bulut Hüner, Nesrin Demir, Mehmet Fatih Kaya

**Affiliations:** † Erciyes University, Engineering Faculty, Energy Systems Engineering Department, Heat Engineering Division, 38039Kayseri, Turkey; ‡ Erciyes University, Graduate School of Natural and Applied Sciences, Energy Systems Engineering Department, 38039Kayseri, Turkey; § Erciyes University H2FC Hydrogen Energy Research Group, 38039Kayseri, Turkey; ∥ BATARYASAN Enerji ve San. Tic. Ltd. Şti., Yıldırım Beyazıt Mah., Aşık Veysel Bul., ERÜ TGB Kuluçka Merkezi, No: 63/B, 38039Kayseri, Turkey

## Abstract

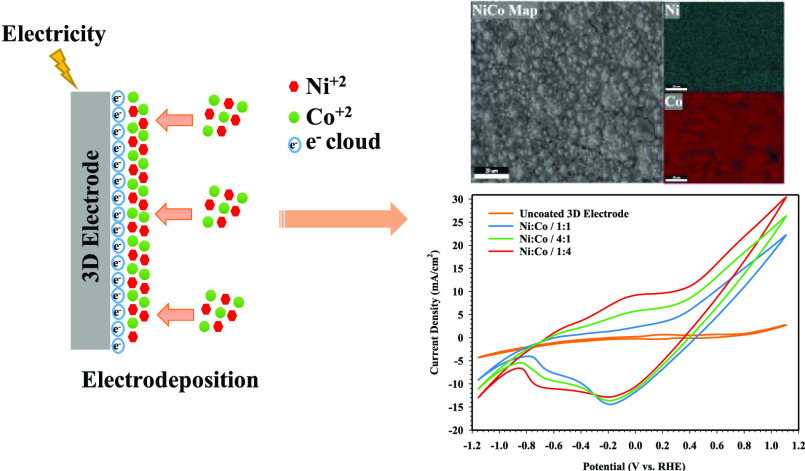

Additive manufacturing has been a very promising topic in recent years for research and development studies and industrial applications. Its electrochemical applications are very popular due to the cost-effective rapid production from the environmentally friendly method. In this study, three-dimensional (3D) printed electrodes are prepared by Ni and Co coatings in different molar ratios. Different Ni/Co molar ratios (*x*:*y*) of the Ni/Co/*x*:*y* alloys are prepared as 1:1, 1:4, and 4:1 and they are named Ni/Co/1:1, Ni/Co/4:1, and Ni/Co/1:4, respectively. According to the results, when the 3D electrode samples are coated with Ni and Co at different molar ratios, the kinetic performance of the NiCo-coated 3D electrode samples for hydrogen evolution reaction is enhanced compared to that of the uncoated 3D electrode sample. The results indicate that the Ni/Co/1:4-coated 3D electrode has the highest kinetic activity for hydrogen evolution reactions (HERs). The calculated Tafel′s slope and overpotential value (η_10_) for HER are determined as 164.65 mV/dec and 101.92 mV, respectively. Moreover, the Ni/Co/1:4-coated 3D electrode has an 81.2% higher current density than the other electrode. It is observed that the 3D printing of the electrochemical electrodes is very promising when they are coated with Ni–Co metals in different ratios. This study provides a new perspective on the use of 3D printed electrodes for high-performance water electrolysis.

## 1 Introduction

To date, energy requirements of the people are mostly met with fossil-based fuels like oil, natural gas, and coil. However, the burning of these fuels leads to serious environmental problems such as global warming, water/air pollution, and harmful gases (CO, CO_2_, NO*_x_*, etc.). Among these gases, approximately 70% of CO_2_ released into the environment causes the greenhouse effect. Therefore, it is necessary to explore new, clean, and sustainable energy sources to reduce the harmful gases caused by fossil fuels.^1−4^ To meet the energy demand due to industrialization, rapid urbanization, and continuous population growth globally, especially in developing countries, many researchers have turned their way to clean and environmentally friendly renewable energy sources. The most common alternative and renewable energy sources are mainly solar,^5^ wind,^6^ geothermal,^7^ biomass,^8^ and hydrogen energies.^9^ Among these resources, hydrogen is regarded as a secondary energy source.^10^ In addition, it is considered the energy carrier of the future due to its many advantages like long-term storage, high energy density, and low environmental impact.^11^ Hydrogen contributes to different forms of energy, such as generating electricity via fuel cells and fuel for gas turbines or internal combustion engines. Hydrogen can be generated from many sources using different methods and technologies, including fossil fuels and renewable energy sources.^12,13^ Global demand for hydrogen is generated by approximately 48% of natural gas, 30% by heavy oils and naphtha, 18% by coal, and the remaining 4% is produced by the electrolysis method of water.^14−17^ Among these methods, hydrogen production via electrolysis of water has attracted the attention of many researchers in recent years due to its important advantages in both alkaline and acidic environments.^18−22^ However, the overpotential in the electrodes, which is an important issue in water electrolysis, is a disadvantage due to high energy consumption and production costs. These disadvantages can be solved by the development of new cathode and anode electrode materials with high electrocatalytic activity, high corrosion resistance, low overpotential, and a large active surface area in hydrogen evolution reactions (HERs) and oxygen evolution reactions (OERs).^23−25^ The development of new and low-cost electrode materials with high kinetic activity and good electrical conductivity is important to reduce energy losses in electrochemical applications. Recently, additive manufacturing (AM), known as the three-dimensional (3D) printing method, has focused the attention of many researchers on producing electrodes due to its many advantages like cost-effectiveness, rapid prototyping, and simple processing properties. In addition, the AM method has shown great potential to reduce energy consumption and wastage materials. It has been stated that the widespread use of this method would lead to a significant reduction of global energy demand by 27%.^26^ Due to the low cost of polymeric filaments and printing devices in the 3D printing process, fused deposition modeling (FDM), which is known as fused filament fabrication (FFF), is commonly utilized by researchers. In the FDM method, polymer-based thermoplastic materials and carbon-based materials are widely used as electrode materials in electrochemical applications.^27,28^ The use of products prepared using conductive filaments in the 3D printed method in electrochemical applications has emerged as an innovative approach. In particular, conductive carbon filaments are obtained from conductive materials such as graphene, carbon nanotubes, and carbon black (CB) mixed with polymeric materials like acrylonitrile butadiene styrene (ABS) and polylactic acid (PLA).^29−32^ Although carbon composite filaments have significant advantages (the low cost and easy method, among others) for producing conductive electrodes using the 3D printing method, the electrical resistance of carbon-based filaments is relatively high. For instance, the carbon black-based Proto-Pasta PLA filament has an electrical resistance of 10.5–12 Ω·cm, while the graphene-based Black Magic PLA filament has an electrical resistance of 0.8–1.2 Ω·cm.^33,34^ To improve the electrical conductivity and kinetic activities of 3D printed electrodes, which are produced from the conductive PLA filament, the electrochemical coating process is required. This process is considered an easy, cost-effective, and highly controllable method for preparing alloy and composite coatings. Moreover, it can be adjusted by controlling the coating parameters like current density, electrolyte composition, pH, bath type, and temperature.^35^ Extensive research in recent times has focused on the use of low-cost transition metals like Ni, Co, and their alloys for the electrolysis of water due to their high catalytic activity and high stability. Among these metals, Ni is the best catalyst for the HER due to its high kinetic activity, long-term stability, and excellent corrosion resistance. It has also been shown that combining Ni with Co or Cu results in significant developments in electrode performance due to low overpotential and high charge transfer. For example, Darband et al.^36^ prepared Ni–Co alloy nanocones by electrodeposition method in the bath solution containing a crystal modifier, and their electrocatalytic properties for HER were investigated in alkali medium (1 M KOH). They stated that the electrocatalytic activities of Ni–Co alloy nanocones are promising, especially for hydrogen production in an alkaline environment. In addition, they emphasized that the nanocone structure can be used as a new structure in electrocatalytic applications for the HER. In another study, Hong et al.^37^ deposited NiCo electrochemically on Cu substrates and investigated their electrocatalytic activities for the HER. They mentioned that the surface morphology of Ni–Co alloys changed with an increase in the Co content and found that the HER performances of Ni–Co alloys are dramatically increased in comparison to those of Ni and Co, and the Ni_49_Co_51_ alloy catalyst showed the highest kinetic activity for the HER. Lupi et al.^38^ deposited Ni–Co alloys electrochemically with Co concentrations ranging from 1.5 to 100% on the Al substrate. They investigated the electrocatalytic performance of NiCo alloys for the HER in an alkaline medium. They noted that the hydrogen overpotential is lower in the case of Co concentrations ranging from 41 to 64% wt. Xue et al.^39^ prepared NiCo coating on a carbon steel sample by varying parameters such as applied potentials and deposition times. They investigated the electrochemical properties of NiCo alloys. They stated that electrochemical measurements showed excellent corrosion inhibition ability of NiCo coating for carbon steel sample at 3.5% wt. solutions. However, according to the authors’ knowledge, there is no study that investigates the effect of HER performance of NiCo coating on 3D printed polymeric electrodes in alkaline media. We have studied the performance of polymeric 3D printed electrodes, which are electrochemically coated by the NiCo alloy. Therefore, the present study aims to evaluate the HER performance of NiCo-coated 3D electrodes with different molar ratios of Ni and Co contents in an alkaline medium. The physical features of NiCo-coated 3D electrode samples (surface morphologies, elemental compositions, crystalline structures, etc.) are measured by using field emission scanning electron microscopy (FE-SEM), FE-SEM–energy-dispersive X-ray spectroscopy (FE-SEM/EDX), FE-SEM mapping, transmission electron microscopy (TEM), X-ray diffraction (XRD), and X-ray photoelectron spectroscopy (XPS) technique. The electrochemical and electrocatalytic properties of Ni–Co-coated 3D electrodes have been measured by using linear sweep voltammetry (LSV), cyclic voltammetry (CV), electrochemical impedance spectroscopy (EIS), Tafel polarization analysis, and chronoamperometry (CA) techniques.

## 2 Experimental Procedure

### 2.1 Materials and Methods

To prepare coating bath solutions of different concentrations, nickel sulfate hexahydrate (NiSO_4_·6H_2_O CAS Number: 10101-97-0), cobalt (II) sulfate heptahydrate (COSO_4_·7H_2_O CAS Number: 10026-24-1), and boric acid (H_3_BO_3_ CAS Number: 10043-35-3) were purchased from Merck, Germany. The commercially available conductive graphene-based polylactic acid (PLA) filament (volume resistivity: 0.6 Ω·cm) for 3D printing of the electrode samples was purchased from Black Magic 3D. All bath solutions were prepared with highly purified deionized (DI) water (resistivity:18.25 MΩ·cm) provided by the water purification system (Milli-Q).

### 2.2 3D Printing of Electrodes Using Conductive PLA Filament

**Figure 1:**
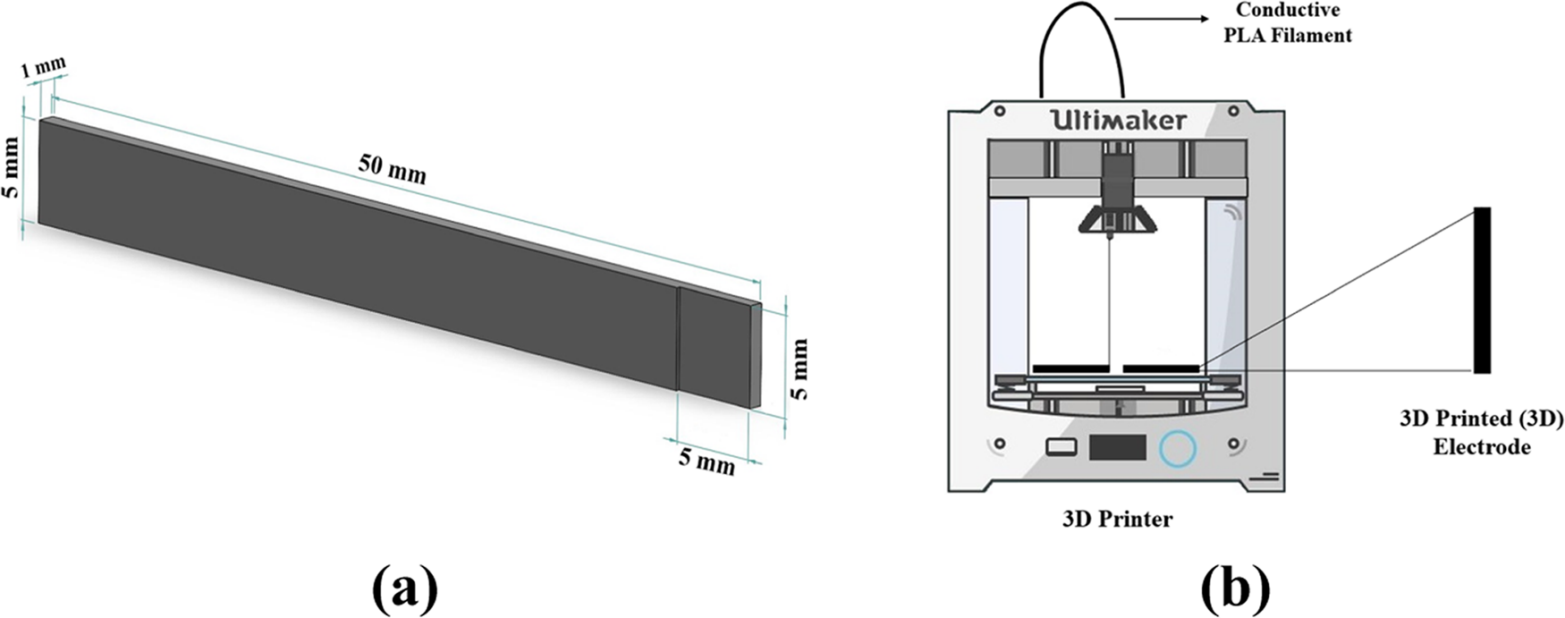
3D electrode design: (a) dimensions of the electrode samples and (b) schematic view of the 3D printing process.

Electrode samples were designed using SolidWorks drawing software with 50 mm (length) × 5 mm (width) × 1 mm (thickness) dimensions. A 3D printer (Ultimaker^2+^) with a 0.4 mm diameter extrusion nozzle was used to prepare electrodes. Then, the designed files were converted into the Standard Triangle Language (*.STL) file format and sliced in the Cura 2.0 software slicer program. For the printing process of the electrode samples, the bed temperature, nozzle temperature, printing speed, and layer thickness were set to 60, 200 °C, 30 mm/s, and 0.1 mm, respectively. All electrodes were printed at 100% infill.^40^ The dimensions of the electrodes for the 3D printing process can be seen in Figure 1. Moreover, the figures of the 3D printed electrodes are presented in Figure S1.

### 2.3 Electrodeposition of 3D Electrodes

For the electrochemical coating process, the active surface area of the 3D printed electrodes was adjusted to 25 mm^2^. The remaining surfaces of the 3D electrode were insulated with Teflon tape. Before the electrochemical deposition, the surface of the 3D electrodes was cleaned with ethanol and pure water. The coating process was conducted by applying a constant voltage with a potentiostat/galvanostat system (Metrohm-Autolab PGSTAT 204) using the two-electrode method. The 3D electrode was used as a cathode, while the Pt sheet with a surface area of 1 cm^2^ was used as an anode. The electrochemical deposition process of Ni/Co/*x*:*y* alloys was carried out under a constant voltage of 10 V for 3600 s.^41^ To prepare the coating bath solution, high-purity water provided by the Milli-Q system was utilized. All of the coating baths were prepared as 50 mL of volume, and experiments were carried out at room temperature (298.15 K). The molar ratios of Ni/Co (*x*:*y*) in the coating bath for alloying samples were prepared as 1:4, 1:1, and 4:1.^42,43^ Then, Ni/Co/*x*:*y* coated 3D electrodes were denoted as Ni/Co/1:1, Ni/Co/1:4, and Ni/Co/4:1. The coating process was stirred with a magnetic stirrer (speed: 300 rpm), and the coating was conducted for 1 h. Molar ratios of the coating baths are given in Table 1.

**Table 1:** Compositions of the Coating Bath Solution Parameters for Ni/Co/*x*:*y* Alloys

	concentration (mol/L)		
bath composition	Ni/Co/1:4 (M)	Ni/Co/1:1 (M)	Ni/Co/4:1 (M)
NiSO_4_·6H_2_O	0.125	0.5	0.5
CoSO_4_·7H_2_O	0.5	0.5	0.125
H_3_BO_3_	0.5	0.5	0.5

The amount of mass deposited on NiCo-coated 3D electrodes was computed from eq 1 using Faraday′s law
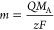
1where *m* is the amount of mass deposited on the electrode; and *Q*, *z*, *M*_A_, and *F* represent the amount of charge passed per unit time, the number of electrons in an electrochemical reaction, the molar weight of the deposited compound (g/mol), and Faraday′s constant (96.485 C/mol), respectively.

### 2.4 Determination of the Physical Properties of 3D Electrodes

The surface morphologies of the 3D electrode samples were analyzed by the FE-SEM device (Zeiss Gemini 500). The elemental analysis of the samples was determined utilizing EDX analyses connected with FE-SEM. The surface micrographs of NiCo-coated 3D electrodes were carried out using a Hitachi High-tech HT7700 high-resolution TEM operating at 200 kV. The crystalline structure of 3D electrode samples was conducted using XRD with Cu Kα radiation (Bruker AXS-D8). The XRD patterns were conducted at a scan range from 0 to 90° with a step size of 0.02°. The average particle size of the 3D electrodes was calculated by using the Scherrer equation,^44^ as given in eq 2
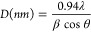
2where *D* is the grain size (nm), and λ, β, and θ represent the X-ray wavelength (λ: 1.54056 Å for Cu), full width at half-maximum of peaks, and peak diffraction angle in radians, respectively. Moreover, the chemical structure of NiCo-coated 3D electrodes was analyzed with high-resolution XPS using a Thermo Scientific Al Kα with an energy of 1000 eV.

### 2.5 Electrochemical Measurements of NiCo-Coated 3D Electrodes

The HER performance of the NiCo-coated 3D electrode samples was measured utilizing LSV, CV, and EIS techniques. The electrochemical experiments were conducted using an Autolab (PGSTAT 204 with FRA32M Module) potentiostat/galvanostat equipment with a conventional three-electrode electrochemical cell. The 3D electrode was used as the working electrode. Ag/AgCl (3 M saturated KCl) and Pt wire were utilized as the reference electrode and counter electrode, respectively. The applied potentials in all electrochemical measurements were calibrated to the reversible hydrogen electrode (RHE) by eq 3
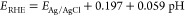
3where *E*_RHE_ is the potential referred to as RHE and *E*_Ag/AgCl_ is the measured potential against the Ag/AgCl (3 M KCl-saturated) reference electrode. LSV analyses were conducted at a scan rate of 5 mV/s between −2 and 0 V vs the Ag/AgCl (3 M KCl-saturated) reference electrode. CV measurements were conducted in the potential range from −1.4 to 0.9 V vs Ag/AgCl (3 M KCl) reference electrode at a scan rate of 10 mV/s.^36,42^ EIS measurements of 3D electrodes were made with the open circuit potential (OCP) with a frequency range between 10 kHz and 0.01 Hz using a 10 mV bias potential. Tafel polarization curves were recorded from −2 to 0.2 V vs Ag/AgCl at a scan rate of 10 mV/s. The long-term stability tests of electrodes were performed with CA analysis at a −0.15 potential for 20 h.^44^ Tafel polarization measurements are used to compare the kinetic activity of the NiCo-coated 3D electrodes. The kinetic parameters of the 3D electrodes for HER are derived from the Tafel eq 4(45)
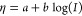
4where η (mV), *I* (mA/cm^2^), *a*, and *b* represent the overpotential, current density, anodic, and cathodic Tafel slopes (mV/dec), respectively. Moreover, the exchange current density (*J*_o_) and the transfer coefficient (*a*) are determined by extrapolating Tafel polarization curves using eqs 5 and 6, respectively, as given below
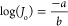
5
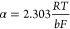
6where *T*, *R*, and *F* represent the temperature in Kelvin (K), the gas constant (*R*: 8.314 J/K/mol), and Faraday′s constant (*F*: 96.485 C/mol), respectively. The formation of hydrogen (H_2_) molecules on the surface of electrodes takes place through multistage electrochemical processes. Basically, the mechanisms of the HER in the alkaline medium are expressed by the following steps.^46^

#### 2.5.1 Volmer Reaction

The electrochemical discharge of the proton on the electrode surface
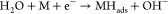
7

#### 2.5.2 Heyrovsky Reaction

The electrochemical desorption of hydrogen
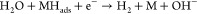
8

#### 2.5.3 Tafel Reaction

The chemical desorption with the combination of adsorbed hydrogen atoms
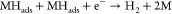
9where M and MH_ads_ represent the electrode surface and the hydrogen adsorbed on the electrode surface, respectively. It should be known that the strength of the H_2_O and MH_ads_ interactions plays a major role in the reaction mechanism and the kinetics of HER. HER reaction starts with the discharge reaction of the proton and the constitution of the hydrogen atom adsorbed on the surface (Volmer reaction). The formation stage of the HER reaction follows the electrochemical desorption of hydrogen (Heyrovsky reaction) or the chemical desorption of hydrogen from the electrode surface (Tafel reaction).^47^ All measurements were conducted in a 1 M KOH solution held in N_2_ gas during the experiments.

## 3 Results and Discussion

### 3.1 Surface Morphology, Elemental Composition, and Crystalline Structure of 3D Electrodes

**Figure 2:**
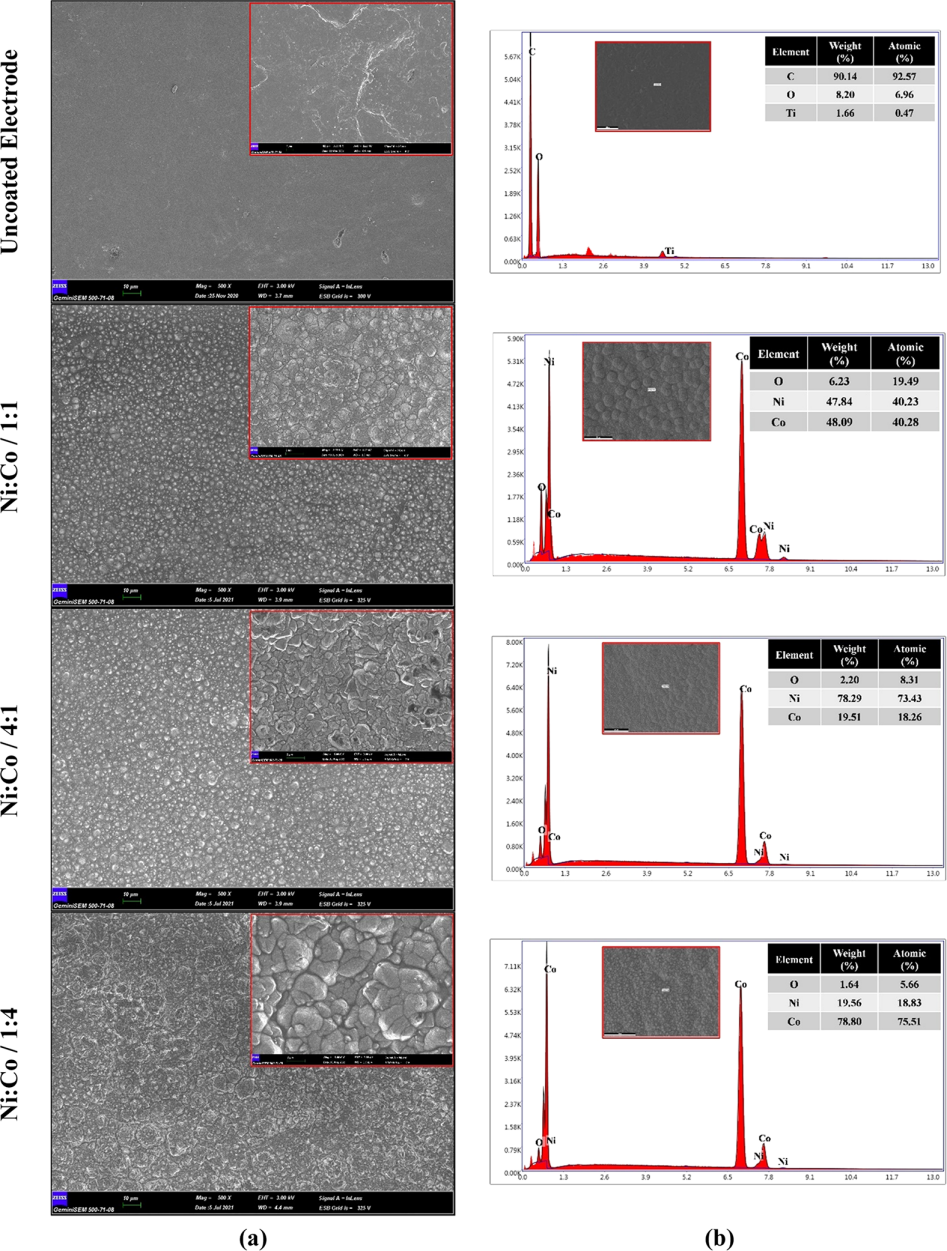
(a) FE-SEM images and (b) FE-SEM–EDX spectra of NiCo-coated 3D electrode samples.

FE-SEM images and FE-SEM–EDX results of uncoated and NiCo-coated 3D electrode samples can be seen in Figure 2. Figure 2a shows FE-SEM images of 3D electrodes. In addition, the FE-SEM–EDX spectra of samples can be seen in Figure 2b. As seen in Figure 2a, it is seen that there is no change in the surface of the uncoated electrode without any coating process. However, when the electrode samples are coated with Ni and Co in different molar ratios, it is determined that NiCo coatings with a granular structure are observed in the surface morphology of the electrodes. Moreover, it is observed that NiCo-coated 3D electrodes have a granular structure containing grains of different sizes depending on their chemical composition. Therefore, the surface morphology and grain size of the NiCo-coated 3D electrodes are based on the concentration of Ni and Co in the coating solution. Moreover, according to the FE-SEM images, the clusters of particles are observed on the surface of the NiCo-coated 3D electrodes, which may be attributed to the synergistic interactions of Ni and Co particles. Depending on the crystal structure of the particles, the particle sizes on the surface of the electrodes can be different from the uniformity of the clusters of particles. These clusters of particles can provide more active sites and a larger catalytic active surface area, allowing ions to diffuse and transport faster. This accelerates the HER reactions of NiCo-coated 3D electrodes.^36^ According to Faraday′s law in eq 1, the amounts of deposited mass on the Ni/Co/1:1-, Ni/Co/4:1-, and Ni/Co/1:4-coated 3D electrodes are calculated as 0.0320, 0.0376, and 0.0429 g/cm^2^, respectively. When the amount of Ni and Co is increased in the coating bath, it is determined that the grain size becomes larger in the NiCo-coated 3D electrodes (as given in Figures 2 and 4). At the same time, it is seen that the granular structures deposited on the electrode surface increase with the increase of the Co content (Figure 2a).

**Figure 3:**
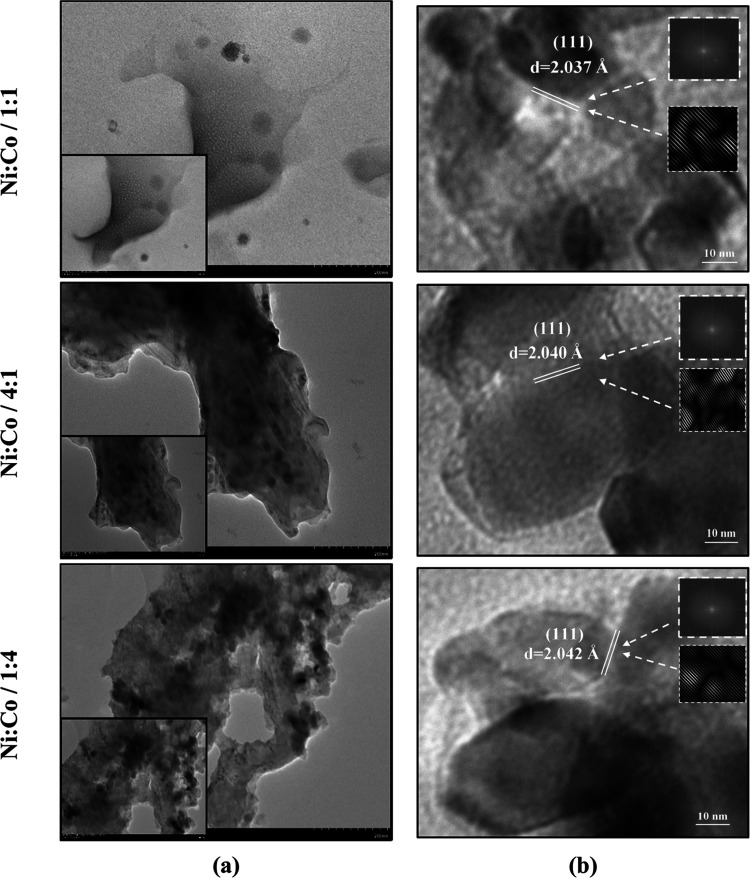
TEM images and high-resolution TEM images of NiCo-coated 3D electrodes: (a) TEM image and (b) FFT patterns.

As seen in Figure 2b, the FE-SEM/EDX analysis of the uncoated 3D electrode, the weight percentage ratio of elements obtained as C, O, and Ti is 90.14, 8.20, and 1.66, respectively. Ti assets in the uncoated sample are due to the inherent metal impurity in the graphene-based PLA filament.^48,49^ After the coating process, characteristic peaks in the FE-SEM/EDX analysis of the NiCo-coated 3D electrodes consist of Co, Ni, and O elements. Ni and Co peaks are mainly composed of codeposited NiCo alloys. The O element′s peak might be formed due to the oxidation of both Ni and Co particles in contact with humidity and O_2_ in the air. The presence of Ni and Co peaks is revealed in different relative intensities on the surface of the polymer electrodes owing to the different compositions of the coating bath. Whereas the highest Co content is obtained with 78.80% wt. in the Ni/Co/1:4-coated 3D electrode, the highest Ni content is obtained with 78.29% wt. in the Ni/Co/4:1-coated 3D electrode. It is also seen that the weight percentages of Ni (47.84% wt.) and Co (48.09% wt.) contents in the Ni/Co/1:1-coated 3D electrode are almost the same. According to FE-SEM-EDX analysis, it has been proved that the composition of NiCo coatings is uniform by electrochemical deposition. Figure S2 shows the color mapping analysis of the NiCo-coated 3D electrodes to determine the elemental distribution. The elemental FE-SEM mapping images show a homogeneous distribution of Ni (green) and Co (red) elements throughout the surfaces of the NiCo-coated 3D electrodes, which confirm Ni and Co presence by electrodeposition on the surface of the electrodes. Crystal structure and microstructure of NiCo-coated 3D electrodes are investigated by using TEM analysis. A bright field in TEM micrographs for different sizes of NiCo-coated 3D electrode samples can be seen in Figure 3.

**Figure 4:**
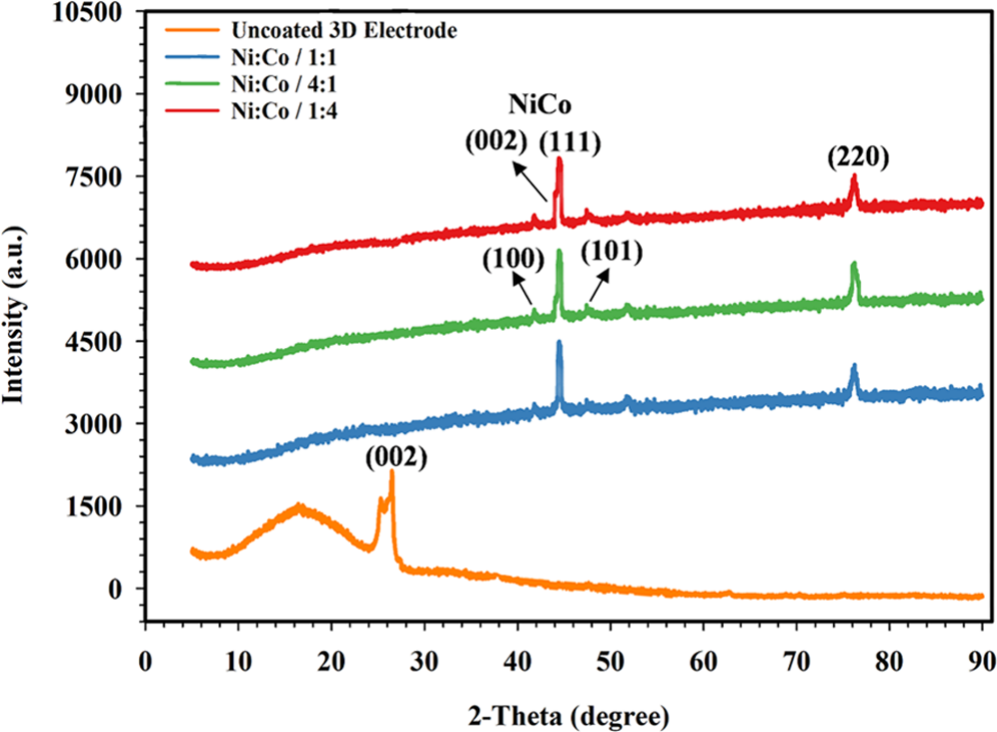
XRD patterns of prepared NiCo-coated 3D electrode samples.

Figure 3a shows TEM images of NiCo-coated 3D electrodes, and Figure 3b represents a high-resolution view of a certain region from Figure 3a. Figure 3b shows a Fourier transform (FFT) diagram of a particular region. As can be seen from TEM images of the electrode samples (Figure 3a), the surface of the electrodes exhibits a sheet-like morphology. The distance between the lattice fringes of the images is obtained from the TEM analysis, and the hkl planes of the crystal structure corresponding to a *d*-spacing value are determined by using ImageJ software program.^50^ As can be seen in the high-resolution image (Figure 3b), the lattice fringes with a d-spacing of NiCo-coated electrodes are calculated as 2.037, 2.040, and 2.042 Å, respectively, which corresponds to the (111) crystal plane of the cubic phase NiCo alloys. The smaller lattice fringe of the Ni/Co/1:1-coated 3D electrode may be due to its smaller particle sizes compared to that of other 3D electrodes. It is concluded that the *d*-spacing value is compatible with the plane distances of the *hkl* (111) crystal structure of the NiCo alloy, which has a face-centered cubic (FCC) structure as given in XRD results (Figure 4). In Figure 4, XRD patterns of the NiCo-coated 3D electrodes coated with an electrolyte containing different molar ratios of Ni and Co can be seen. The diffraction peaks corresponding to NiCo alloys have occurred in 3D-coated electrodes. As seen in Figure 4, a crystalline peak, which corresponds to the (002) graphite peak, is observed at 2θ = 26.428° in the XRD pattern of the uncoated electrode (JCPDS card number: 00-012-0212).^51^ However, it is seen that there are NiCo peaks on the surface of the 3D electrodes coated with different molar ratios, and the intensity of graphite peaks is very low. It is determined that the diffraction peaks of the NiCo-coated 3D electrode samples in the XRD patterns have matched the diffraction peaks of the Ni and Co phases. When Figure 4 is examined, the XRD peaks (JCPDS Card No: 96-152-5375) at 43.96, 44.24, 51.87, and 76.17° consist of NiCo planes like (002), (111), (200), and (220), respectively. Ni and Co have hexagonal close-packed (HCP) and face-centered cubic (FCC) structures depending on the chemical composition of the NiCo alloy.^52^ The results indicated that the NiCo-coated 3D electrodes have an FCC phase in the orientation of (111) and (220).

The Ni and Co coatings have a similar crystal formation. In addition, the mechanical and kinetic activity features of pure Ni could be improved by alloying it with the Co element. NiCo-coated samples exhibit high wear resistance and mechanical strength, and they are highly corrosion-resistant due to the low corrosion potential of Co.^53,54^ Grain size is one of the important properties for the kinetic activity of the metals as well as NiCo coatings because NiCo coatings with higher Co and Ni contents exhibit a high grain size.^55^ Phase structure and grain size of NiCo-coated 3D electrodes are listed in Table 2. According to Table 2, the size of grains in the (111) phase of NiCo-coated 3D electrodes is increased depending on the Ni and Co contents. Also, there are peaks of Co in the HCP phase ((100) and (101), JCPDS Card No: 96-901-1616), which explains the formation of different phases of Co in the NiCo-coated 3D electrodes. As can be seen in the XRD pattern, with a higher composition of Co in the electrode surface, it causes an increase of the hcp diffraction peak intensity. This situation may be relevant to the different phase separations of amorphous Ni or Co. Thus, the increase in the Co content in the coating bath solution could enhance the number of active sites and this may improve the kinetic activity and HER performance of the electrode specimens.

**Table 2:** Phase Structure and Grain Size of NiCo-Coated 3D Electrodes

electrode	FWHM (deg)	*d* (Å)	β	plane (*hkl*)	phase structure	grain size (nm)
Ni/Co/1:1	44.368	2.040	0.328	(111)	FCC	26.80
Ni/Co/4:1	44.269	2.044	0.326	(111)	FCC	27.03
Ni/Co/1:4	44.468	2.035	0.312	(111)	FCC	28.26

**Figure 5:**
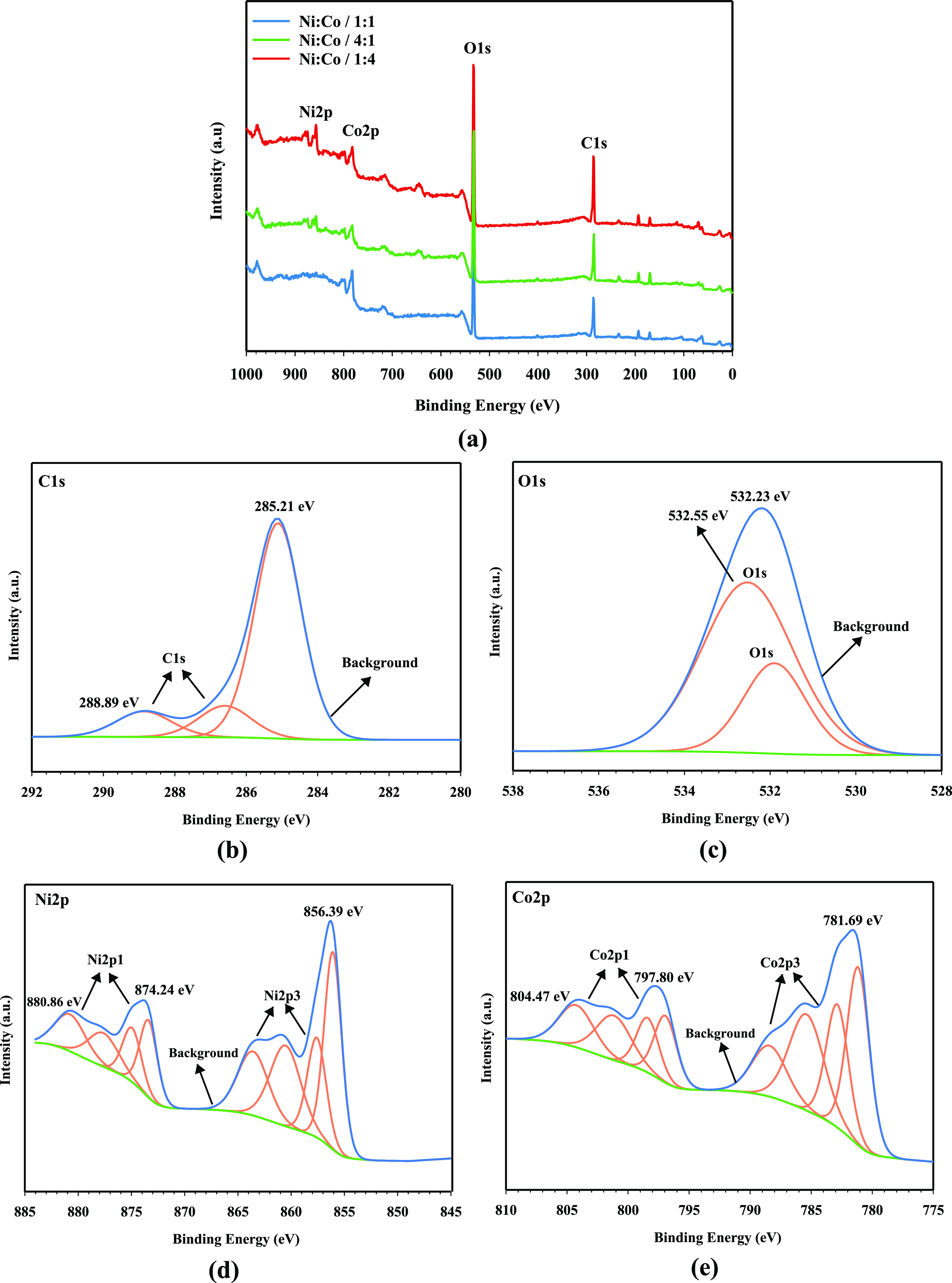
(a) XPS survey patterns of NiCo-coated 3D electrodes and detailed XPS spectra of the Ni/Co/1:4 electrode: (b) C 1s, (c) O 1s, (d) Ni 2p, and (e) Co 2p.

The surface chemistry of the NiCo-coated 3D electrodes has been performed by using XPS analysis. The XPS survey of the electrode samples can be seen in Figure 5. On the other hand, the detailed XPS spectra for C 1s, O 1s, Ni 2p, and Co 2p of the Ni/Co/1:4-coated electrode are shown in Figure 2b–e. The XPS survey pattern for other coated electrodes (Ni/Co/1:1 and Ni/Co/4:1) can be seen in Figures S3 and S4 (the asset of C, O, Ni, and Co).

**Figure 6:**
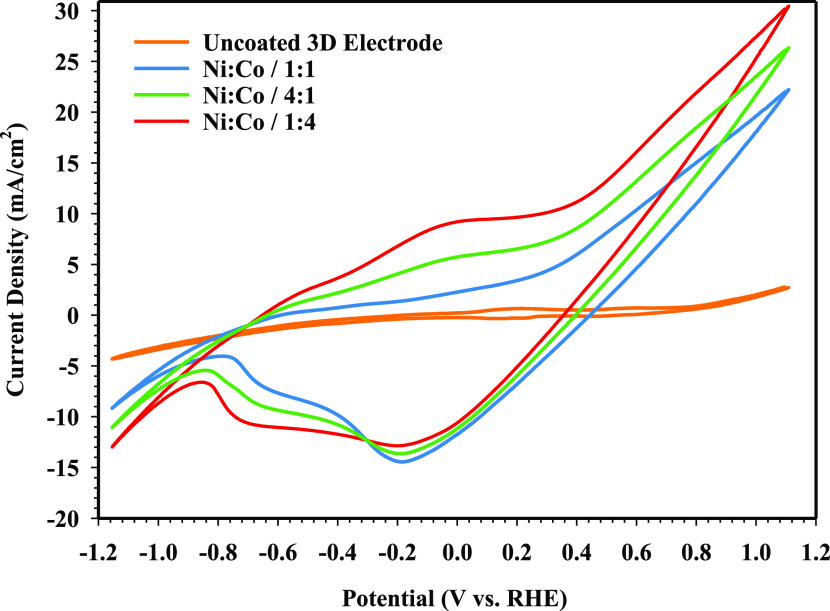
CV measurements of NiCo-coated 3D electrode samples.

XPS survey of NiCo-coated electrodes exhibits the presence of C, O, Ni, and Co on the electrode surface, and these elements are also given in EDX analysis, which is coherent with XPS survey results. As given in Figure 5b, the C 1s spectrum has been assigned to three peaks, indicating C=C/C–C (285.21 eV), C–O (286.65 eV), and C=O (288.89 eV). In Figure 6c, the O 1s spectrum has matched into three peaks, corresponding to C=O (532.23 eV), C–OH (532.55 eV), and C–O–C (531.25 eV).^56^ As can be seen in Figure 6c,6d, the binding energies of metallic Ni obtained at 857.59/875.12 and 856.19/873.98 eV have matched to Ni^2+^ and Ni^3+^. Moreover, the binding energies of Ni^2+^ and Ni^3+^ have two oxidized peaks that can be matched to Ni(OH)_2_ and its satellite peak (NiO), respectively. Whereas the metallic binding energies of Co are assigned to 783.45 and 804.47 eV, indicating the coexistence of Co^2+^ and Co^3+^,^57,58^ the binding energies of Co^2+^ and Co^3+^ correspond to Co(OH)_2_ and its CoO, respectively. The presence of oxidized metals on the surface of NiCo-coated electrodes may be due to partial oxidation of the electrode surface or atmospheric exposure of electrodes during analysis. Therefore, the XPS analysis results of the electrodes are in good agreement with both the FE-SEM/EDX and XRD results. NiCo-coated 3D electrodes have little deviations in their binding energies, and the corresponding data for Ni 2p and Co 2p are listed in Table 3.

**Table 3:** Binding Energies of Ni 2p and Co 2p Obtained from the XPS Pattern for the NiCo-Coated 3D Electrode

	Ni 2p^1^ (eV)	Ni 2p^3^ (eV)	Co 2p^1^ (eV)	Co 2p^3^ (eV)
Ni/Co/1:1	874.09	856.18	797.72	781.63
Ni/Co/4:1	874.17	856.23	797.76	781.65
Ni/Co/1:4	874.24	856.39	797.80	781.69

According to Table 3, the binding energies for Ni 2p^1^ compared to those for the Ni/Co/1:4-coated electrode change as 0.15 and 0.07 eV for Ni/Co/1:1- and Ni/Co/4:1-coated electrodes, respectively. Likewise, the corresponding binding energies for Co 2p^1^ have varied between 0.08 and 0.04 eV. This change in the position of the peaks is owing to the difference in electronegativities of Ni and Co. It may also be useful in the movement of the electronic cloud in energy bonding, which may interact with electrode surface charges in the NiCo alloys of the adsorption of hydroxides for the development of HER.^59^

### 3.2 Electrochemical Properties of 3D Electrodes

The CV results of the NiCo-coated 3D electrodes can be seen in Figure 6. The current density value of the uncoated 3D electrode sample is lower than other 3D electrodes. It is seen that peak currents occur in the anodic and cathodic directions when the electrode samples are coated with Ni and Co. According to the CV results, there is an anodic oxidation peak at about −0.625 and −0.103 V and a cathodic reduction peak at about −0.745 and −0.145 V. Also, the peak current of the Ni/Co/1:4-coated 3D electrode is higher than that of the other electrodes, which is probably due to the greater amount of Co. It is observed that the oxidation peak around −0.625 V corresponds to the conversion of Co(0) to Co(II). The peak at around 0.242 V corresponds to the transformation of Co(II)/Co(III), while the peak at around −0.880 V corresponds to the Co(II)/Co(0) transition. In the scanned potential range, a small oxidation peak at the potential of around 0.3 V is attributed to the oxidation of Ni(II) to Ni(III). On the other hand, the cathodic peak at −0.218 V is related to the Ni(III) to Ni(II) reduction.^60,61^

In the CV measurement of the NiCo-coated 3D electrodes, it is seen that the Ni/Co/1:4 coated with a high cobalt content is a 41.61% higher current density for HER than the other electrodes. In addition, the current density of the Ni/Co/4:1-coated 3D electrode at 1.1 V is 19.67% higher than that of the Ni/Co/1:1-coated 3D electrode and this value is 9.80 times higher than that of the uncoated 3D electrode. As a result, increasing the amount of Ni and Co, the kinetic activity of NiCo-coated 3D electrodes has significantly improved in the alkaline medium for HER because particle distribution, grain size, and Ni/Co concentration on the surface of the electrodes have played an essential role in determining kinetic activity.^62^ The LSV curves of the NiCo-coated 3D electrodes can be seen in Figure 8. The onset potential of the uncoated 3D electrode for HER is about −0.4865 V. The onset potentials for Ni/Co/1:1-, Ni/Co/4:1-, and Ni/Co/1:4-coated 3D electrodes were observed as −0.485, −0.479, and −0.475 V, respectively. When the NiCo-coated 3D electrodes are compared, it is observed that the onset potentials of Ni/Co/1:1- and Ni/Co/4:1-coated 3D electrodes for HER are much higher than that of the Ni/Co/1:4-coated 3D electrode. It is the presence of a high amount of Co in the Ni/Co/1:4-coated 3D electrode that results in a high current density and increased kinetic performance of the electrode. As seen in the LSV curve, the Ni/Co/1:4-coated 3D electrode has a 1.75 times higher current density than the Ni/Co/1:1-coated 3D electrode at −1 V. Moreover, the Ni/Co/1:4-coated 3D electrode has a higher HER kinetic activity than other electrode samples. The current density values of NiCo-coated 3D electrodes for HER at −1 V can be summarized in the following order: Ni/Co/1:4 Ni/Co/4:1 > Ni/Co/1:1 > uncoated electrode. It can be seen that the Ni/Co/1:4-coated 3D electrode with a high Co content exhibits a high current density for HER in an alkaline media and provides better kinetic activity in the region of higher current densities. This means that the NiCo-coated 3D electrode has great potential to be utilized in water electrolysis under alkaline conditions. In addition, it is determined that uncoated, Ni/Co/1:1-, Ni/Co/4:1-, and Ni/Co/1:4-coated 3D printed electrodes have overpotentials of −652.63, −157.51, −110.83, and −101.92 mV at a current density of −10 mA/cm^2^, respectively. On the other hand, the reason why the Ni/Co/1:4-coated 3D electrode has a lower overpotential than the Ni/Co/1:1-coated 3D electrode may depend on the synergistic effect between Ni and Co elements as well as the higher Co content. Thus, among the NiCo-coated 3D printed electrodes, the Ni/Co/1:4-coated electrode exhibits the highest kinetic activity and lowest overpotential with a current density of −10 mA/cm^2^. Moreover, the Ni/Co/1:4-coated 3D electrode both has a good kinetic activity for hydrogen formation at a low current density and has a perfect kinetic activity compared to other electrodes available at high current density.

**Figure 7:**
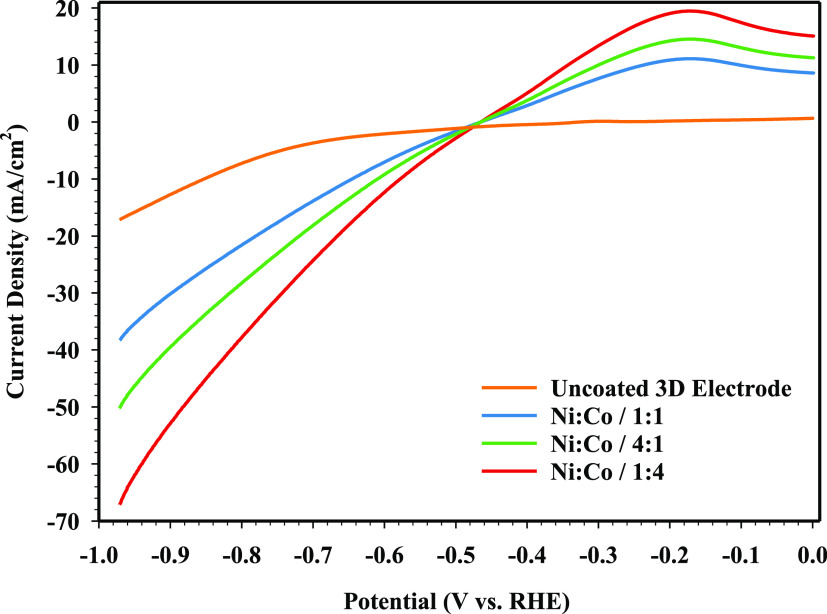
LSV measurements of NiCo-coated 3D electrode samples.

It can be seen in Figure 7 that the kinetic activity of coated 3D printed electrodes with varying concentrations of Ni and Co is different. By increasing the concentration of Co, the overpotential has decreased from −157.51 to −101.92 mV for coated electrodes. Obviously, the uncoated and Ni/Co/1:4-coated electrodes have the lowest and highest kinetic activities compared to other NiCo-coated 3D electrodes, respectively. A comparative study of the electrochemical properties of Ni-, Co-, and NiCo-based electrodes or catalysts is given in Table 4.

**Table 4:** Comparison of the Electrochemical and Kinetic Parameters of Various NiCo-Based Electrodes or Catalysts for HER in an Alkaline Media

electrode/catalyst	alkaline electrolyte	current density (mA/cm^2^)	overpotential (mV)	Tafel slope (mV/dec)	refs
Ni/Co/1:4	1 M KOH	10	101.92	164.65	this study
NiCo	1 M KOH	10	107	120	(36)
NiCo-20 s	1 M KOH	10	152	91	(63)
NiCoP/rGO	1 M KOH	10	209	124.10	(64)
NiCo_2_S_4_	1 M KOH	10	210	81.3	(65)
NiCo/NF	1 M KOH	10	221	137.4	(66)
NiCo_2_S_4_	1 M KOH	10	226	116	(67)
C@NiCo11	1 M KOH	10	232	194	(68)
NiCoTi-3	1 M KOH	10	260	90	(59)
NiCoFe–MOF	0.1 M KOH	10	270	114	(69)
NiCo	0.1 M KOH	10	272.3	114.2	(70)
Co–Ni–G	6 M KOH	10	330	105.3	(71)
Ni_58_Co_42_	1 M NaOH	10	162	60	(72)
NiCo_2.1_P	1 M NaOH	10	175	133	(44)

**Figure 8:**
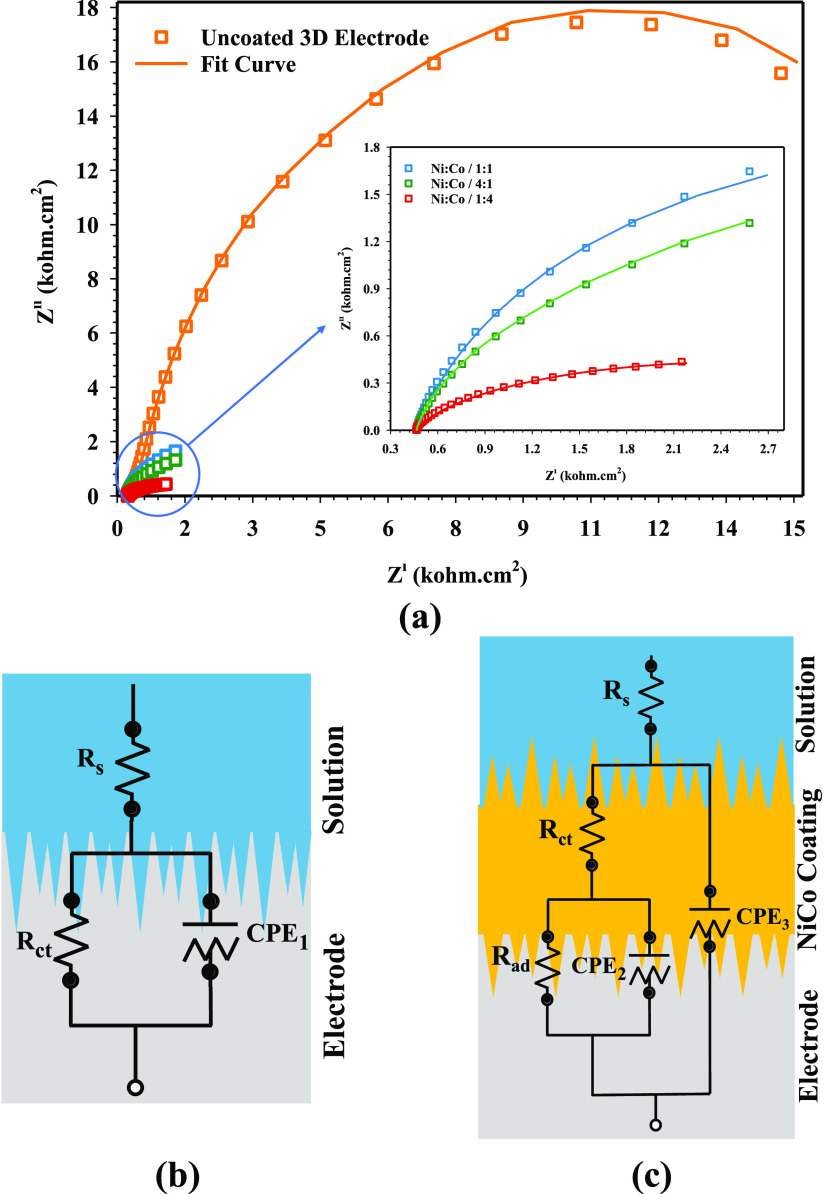
EIS analysis and EEC model of the 3D electrode samples: (a) EIS curves, (b) circuit model of the uncoated 3D electrode sample, and (c) circuit model of NiCo-coated 3D electrode samples.

According to Table 4, the low overpotential (101.92 mV) for the Ni/Co/1:4-coated 3D printed electrode examined at a current density of 10 mA/cm^2^ is very competitive with the recently reported catalyst or electrode, such as NiCo (107 mV),^36^ NiCo-20 s (152 mV),^63^ NiCoP/rGO (209 mV),^64^ NiCo_2_S_4_ (210 mV),^65^ NiCo/NF (221 mV),^66^ and C@NiCo11 (232 mV).^68^ Thus, these data display that NiCo-coated 3D printed electrodes have higher kinetic performance at low overpotentials than the other electrode. Figure 8 shows the EIS results of NiCo-coated 3D electrodes, and Figure 8a shows the Nyquist plot of the 3D electrodes. Figure 8b,8c shows the model of equivalent electrical circuits (EECs) of the uncoated electrode and NiCo-coated 3D electrodes, respectively. The EEC circuit model is composed of elements such as *R*_s_, *R*_ct_, *R*_ad_, and CPE. *R*_s_ represents the solution resistance in the electrolyte (1 M KOH). *R*_ad_ and *R*_ct_ represent hydrogen adsorption resistance and the charge-transfer resistance at the electrode–electrolyte interface, respectively. In this EEC circuit model, CPE_1_, CPE_2_, and CPE_3_ are defined as time constant elements. Whereas CPE_2_ corresponds to the charge-transfer kinetics of the electrodes, CPE_3_ corresponds to hydrogen adsorption for HER kinetics. *n* is the deviation value of the CPE varying from 0 to 1.^39,73^

In the EEC, CPE is utilized instead of *C*_dl_, which is associated with the electrode′s surface structure, and the electrochemically active specific surface area (ECSA) of electrodes can be estimated by EIS fitting results. To determine the ECSA of NiCo-coated 3D electrodes with different deposition ratios, the double layer capacitance (*C*_dl_) is determined by using EIS analysis owing to the fact that *C*_dl_ is proportional to the ECSA
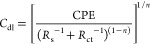
10

The ECSA of the electrodes can be calculated by eq 11 as follows
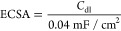
11where *C*_dl_ and *C*_s_ represent the double layer capacitance and specific capacitance, respectively. *C*_s_ of a 1 cm^2^ flat surface area is usually taken as 0.04 mF/cm^2^.^74,75^ The current density normalized by ECSA can be computed by eq 12 as follows
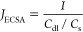
12where *J*_ECSA_, *I*, and *C*_dl_/*C*_s_ represent the normalized current density (mA/cm^2^), current (mA), and ECSA (cm^2^), respectively. The intrinsic activity (τ) of the electrode samples can be determined from the EIS parameter data. The intrinsic activity of the electrodes can be calculated by eq 13 as follows
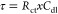
13where τ, *R*_ct_, and *C*_dl_ represent the intrinsic activity, charge-transfer resistance, and double layer capacitance, respectively.^76,77^

The obtained results from the fitting parameters of the EIS curve of electrodes are given in Table 5.

**Table 5:** Fitting Parameters from EIS Analysis Results

electrode	uncoated	Ni/Co/1:1	Ni/Co/4:1	Ni/Co/1:4	estimated error (%)
*R*_s_(kΩ·cm^2^)	0.4775	0.4675	0.4531	0.4725	0.0987
CPE_1_(s*^n^* Ω^–1^ cm^2^)	9.343 × 10^–3^				0.0964
*n*_1_	0.769				0.0970
*R*_ct_(kΩ·cm^2^)	55.075	0.2125	0.2043	0.1910	0.1041
CPE_2_(s*^n^* Ω^–1^ cm^2^)		1.037 × 10^–4^	1.015 × 10^–4^	9.906 × 10^–5^	0.1025
*n*_2_		0.608	0.612	0.625	0.1031
*R*_ad_(kΩ·cm^2^)		5.436	3.8575	1.9775	0.1054
CPE_3_(s*^n^* Ω^–1^ cm^2^)		1.544 × 10^–4^	1.443 × 10^–4^	1.425 × 10^–4^	0.1072
*n*_3_		0.751	0.794	0.84	0.1012
*C*_dl_(F/cm^2^)		0.07146	0.14074	0.29354	
*X*^2^	0.00678	0.01165	0.01127	0.01023	

The parameters obtained from the EIS curves provide information about the kinetic activity for HER, and it varies depending on *R*_ad_ and *R*_ct_. According to Table 5, the solution resistance is almost the same for the electrode samples. As seen in Table 5, the *R*_ct_ and CPE_1_ values of the uncoated 3DE electrode are 55.075 kΩ·cm^2^ and 9.343 × 10^–3^, respectively. It is determined that these values are decreased significantly when the electrode samples are electrochemically coated with Ni and Co. Moreover, the decrease of *R*_ct_ with overpotential shows the enhancement of the HER kinetics. The *R*_ct_ values of Ni/Co/1:1-, Ni/Co/4:1-, and Ni/Co/1:4-coated 3D electrodes in the high-frequency region are measured as 0.2125, 0.2043, and 0.1910 kΩ·cm^2^, respectively. It is seen that the Ni/Co/1:4-coated 3D electrode, which has the lowest *R*_ct_ value, has the highest kinetic activity for HER. The substantial decline in the *R*_ct_ value displays good conductivity and faster charge transfer at the coated electrode and electrolyte interface. The semicircle in the Nyquist plot is related to transfer in the electrode/electrolyte interface, which is controlled by the charge-transfer process. Thus, the Ni/Co/1:4-coated 3D electrode exhibits a similar diameter of the semicircle to the other coated electrodes, which indicates faster charge-transfer kinetics. On the other hand, it is found that the *R*_ad_ values of the NiCo-coated 3D electrode samples decrease with the overpotential. The lowest *R*_ad_ value among the NiCo-coated samples is observed in the Ni/Co/1:4-coated 3D electrode. *X*^2^ and estimated error (%) values have been provided in the manuscript by fitting EIS data of NiCo-coated 3D electrodes with the EEC circuit model (Table 5). It has been determined that a value of *X*^2^ is lower than 0.012, which is accepted as a good approximation criterion to the experimental data, and it represents a deviation less than or equal to 1% according to the obtained data. Moreover, in the EEC circuit model provided from EIS measurements, it is determined that the estimated error rates of the fitting parameters of the EIS curve of electrode samples are below 10%. Thus, it can be assessed to confirm that the proposed EEC model matches the experimental data, and this circuit model is physically reasonable.

As can be seen in Table 5, it is observed that the *C*_dl_ values of the electrodes increased compared to the Co and Ni contents in the coating bath solution. The Ni/Co/1:4-coated 3D electrode exhibits a *C*_dl_ value of 0.29354 F/cm^2^, superior to Ni/Co/1:1- (0.07146 F/cm^2^) and Ni/Co/4:1 (0.14074 F/cm^2^)-coated 3D electrodes. Moreover, a high *C*_dl_ value of electrode samples could also be ascribed to a larger number of sites. Correspondingly, the ECSA of NiCo-coated 3D electrodes is found to be 0.0715, 0.1407, and 0.2935 cm^2^ for Ni/Co/1:1-, Ni/Co/4:1-, and Ni/Co/1:4-coated electrodes, respectively. The ECSA of the Ni/Co/1:4-coated 3D electrode is 0.2935 cm^2^ and higher than other coated electrodes. This state can also be clearly seen in the FE-SEM images. The high kinetic activity of the Ni/Co/1:4-coated 3D electrode may be due to the improvement of ECSA, an increase of active sites for hydrogen adsorption, a high content of Co, and the synergistic effect of Ni and Co. The intrinsic activity has been provided by normalizing all of the coated electrode samples by the ECSA and obtained by a surface area-independent parameter through EIS analysis. Moreover, as τ is inversely proportional to the specific activity, 1/τ can be calculated to compare the intrinsic activity of each electrode obtained using EIS fitting parameters and ECSA-normalized current density. It is determined that the intrinsic activities (eq 13) independent of the area of the Ni/Co/1:1-, Ni/Co/4:1-, and Ni/Co/1:4-coated 3D electrodes have competed as 15.59, 28.76, and 56.06, respectively. According to these results, it is determined that the intrinsic activity of the Ni/Co/1:4-coated 3D electrode in the alkaline media is greater than that of the other coated electrode samples. Moreover, to evaluate the intrinsic activity of the coated electrode samples in detail, we calculated the ECSA-normalized current density of the LSV curves utilizing the ECSA values. The ECSA-normalized HER polarization curve and *J*_ECSA_ values of NiCo-coated 3D electrodes can be seen in Figure 5S. As seen in Figure 5S, after ECSA normalization, *J*_ECSA_ of the Ni/Co/1:4-coated 3D electrode (56.63 mA/cm^2^) is 3.05 times as large as that of the Ni/Co/1:1-coated 3D electrode (18.59 mA/cm^2^) and 1.89 times that of the Ni/Co/4:1-coated 3D electrode (29.81 mA/cm^2^). Therefore, the Ni/Co/1:4-coated 3D electrode exhibits higher *J*_ECSA_ compared to other electrodes at the same test potential for HER. This showed that the activity determined from the current density normalized by ECSA reflects the intrinsic activity of the electrodes and it varies depending on its Ni and Co concentrations in the coating bath. Therefore, the HER activity of the NiCo-coated electrode is not only due to its large ECSA but also closely correlated with its high HER activity, which may be due to the synergistic effect between Ni and Co. Moreover, this could be related to the decreased *R*_ct_ value with increasing Co amount in the coating bath solution, and these results displayed that increasing the amount of Co in the coating bath solution effectively enhances intrinsic activity.

### 3.3 Kinetic Activity and Stability of 3D Electrodes

**Figure 9:**
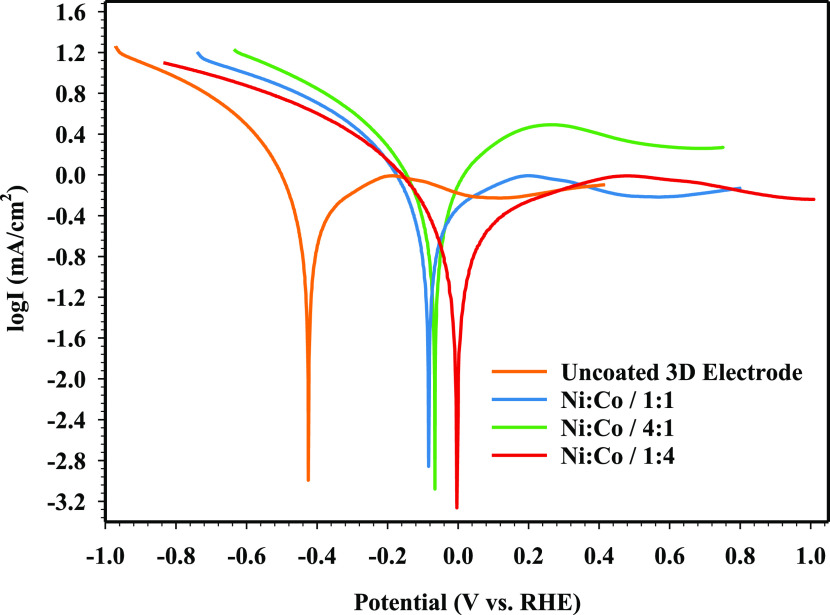
Tafel polarization curves of electrodes in an alkaline media (1 M KOH) at 25 °C.

The Tafel polarization curves give information about the reaction mechanism and reaction rate kinetics of electrodes for HER. The Tafel polarization curves of uncoated, Ni/Co/1:1-, Ni/Co/4:1-, and Ni/Co/1:4-coated 3D electrodes can be seen in Figure 9.

As shown in Figure 9, the uncoated 3D electrode has the lowest kinetic activity for HER by comparison with NiCo-coated 3D electrodes. However, it is seen that the kinetic activities are improved when the electrode samples are coated with different molar ratios of Ni and Co. The kinetic parameters of the electrodes calculated using the Tafel equation are listed in Table 6.

**Table 6:** Tafel Kinetic Parameters of NiCo-Coated 3D Electrodes

electrode	*E*_corr_ (mV)	*I*_corr_(mA/cm)	*b*(mV/dec)	*a*(mV/dec)	α	*J*_o_(mA/cm)
uncoated	–854.32	4.7980 × 10^–4^	380.27	272.36	0.155	1.922 × 10^–4^
Ni/Co/1:1	–839.56	5.6608 × 10^–5^	232.93	157.46	0.253	2.108 × 10^–3^
Ni/Co/4:1	–828.64	4.8381 × 10^–5^	178.61	110.80	0.331	2.396 × 10^–3^
Ni/Co/1:4	–801.83	4.1672 × 10^–5^	164.65	101.92	0.359	2.404 × 10^–3^

**Figure 10:**
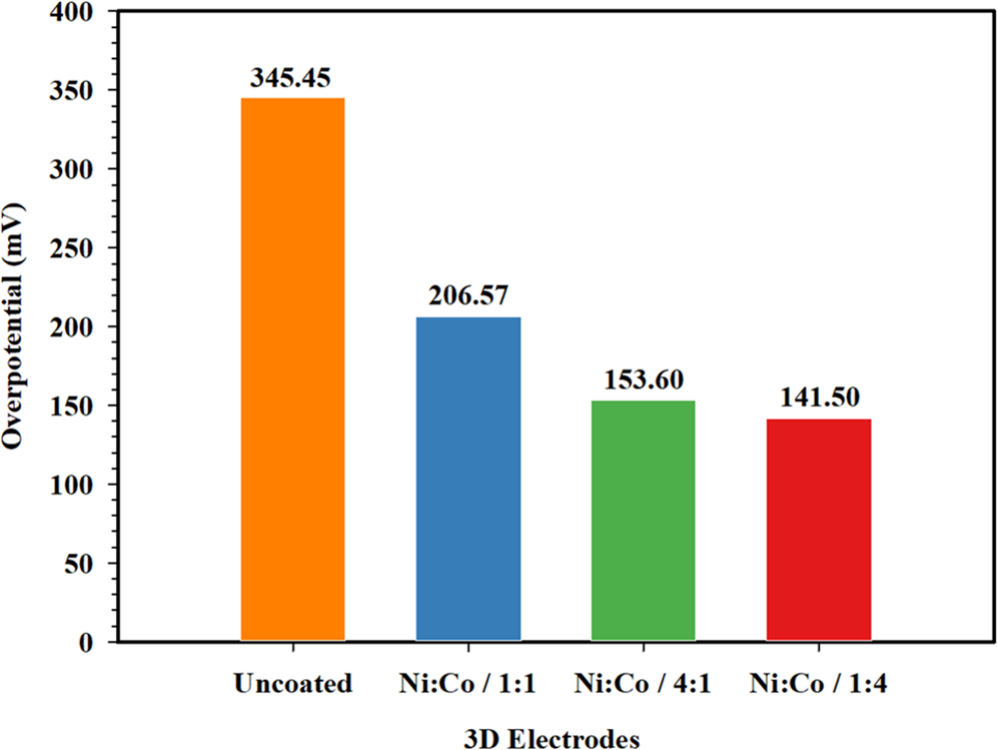
Overpotential values of the 3D electrodes.

According to Table 6, it is seen that the corrosion potential (*E*_corr_) of the 3D electrodes varies between −854.32 and −801.83 mV. The uncoated electrode exhibits the highest Tafel slope (380.27 mV/dec), the lowest exchange current density (1.922 × 10^–4^ mA/cm^2^), and the highest overpotential (345.45 mV). Among all of the NiCo-coated 3D electrodes, the Ni/Co/1:4-coated 3D electrode has exhibited the best HER kinetic activity with a Tafel slope of 164.65 mV/dec in an alkaline medium. A low Tafel slope value is affected by the high charge transfer and the rate of hydrogen adsorption on the surface.^78,79^ It can be clearly seen that cathodic and anodic curves of the NiCo-coated 3D electrodes shifted to lower current densities compared to those of the uncoated 3D electrode sample, and the shift increases with the increase of Ni and Co contents in the coating bath solution. Transfer coefficient (α) is used to compare the effectiveness of NiCo-coated 3D electrodes, and it is expected to have a high electron-transfer coefficient value. The value of the transfer coefficient (α) is widely accepted in determining the HER reaction rate.^80^ As given in Table 6, the α value of the Ni/Co/1:4-coated 3D electrode has higher than that of the uncoated and other NiCo-coated 3D electrodes. The values of the overpotential of electrodes have been calculated by extrapolation of the cathodic slope (*b*) and anodic slope (*a*) of corrosion potential (*E*_corr_), and the results are given in Figure 10. As seen in Figure 10, the uncoated electrode has the highest overpotential of 345.45 mV. It is also clear that the uncoated 3D electrode has a lower kinetic activity than the NiCo-coated 3D electrode samples because of the higher overpotential.

**Figure 11:**
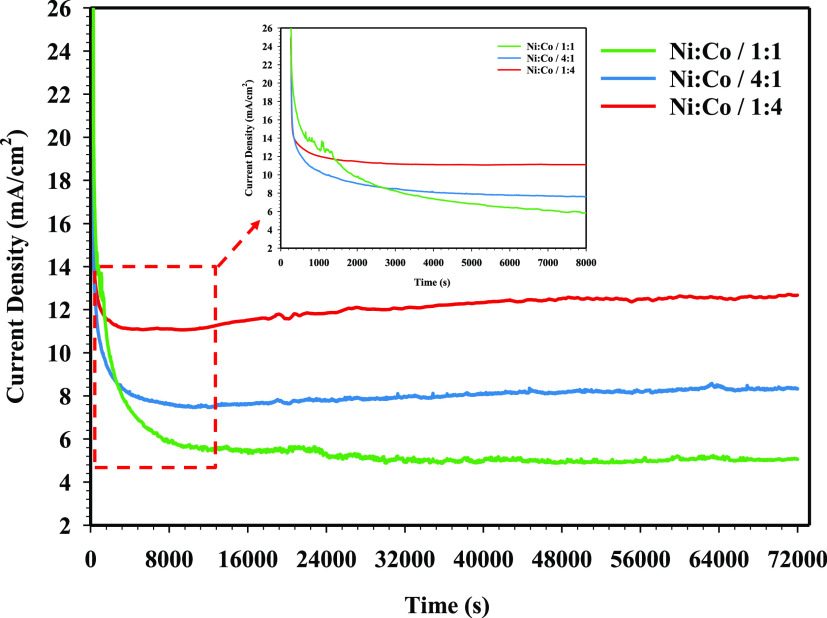
CA measurement results of 3D electrode samples.

It has been determined that when the 3D electrode samples are coated with Ni and Co, the overpotentials of the 3D electrode samples decrease due to the increase of the exchange current density. It is observed that the Ni/Co/1:4-coated 3D electrode shows the lowest overpotential (141.50 mV) when compared to Ni/Co/1:1- (206.57 mV) and Ni/Co/4:1 (153.60 mV)-coated 3D electrode samples. These results indicate that the Ni/Co/1:4-coated 3D electrode provides higher kinetic activity and a great HER performance compared to other 3D electrode samples. The electrocatalytic stability of NiCo-coated 3D electrodes has been conducted by using CA analysis at −0.15 V potential for 20 h in a 1 M KOH solution, and the stability results of the electrodes are given in Figure 11.

**Figure 12:**
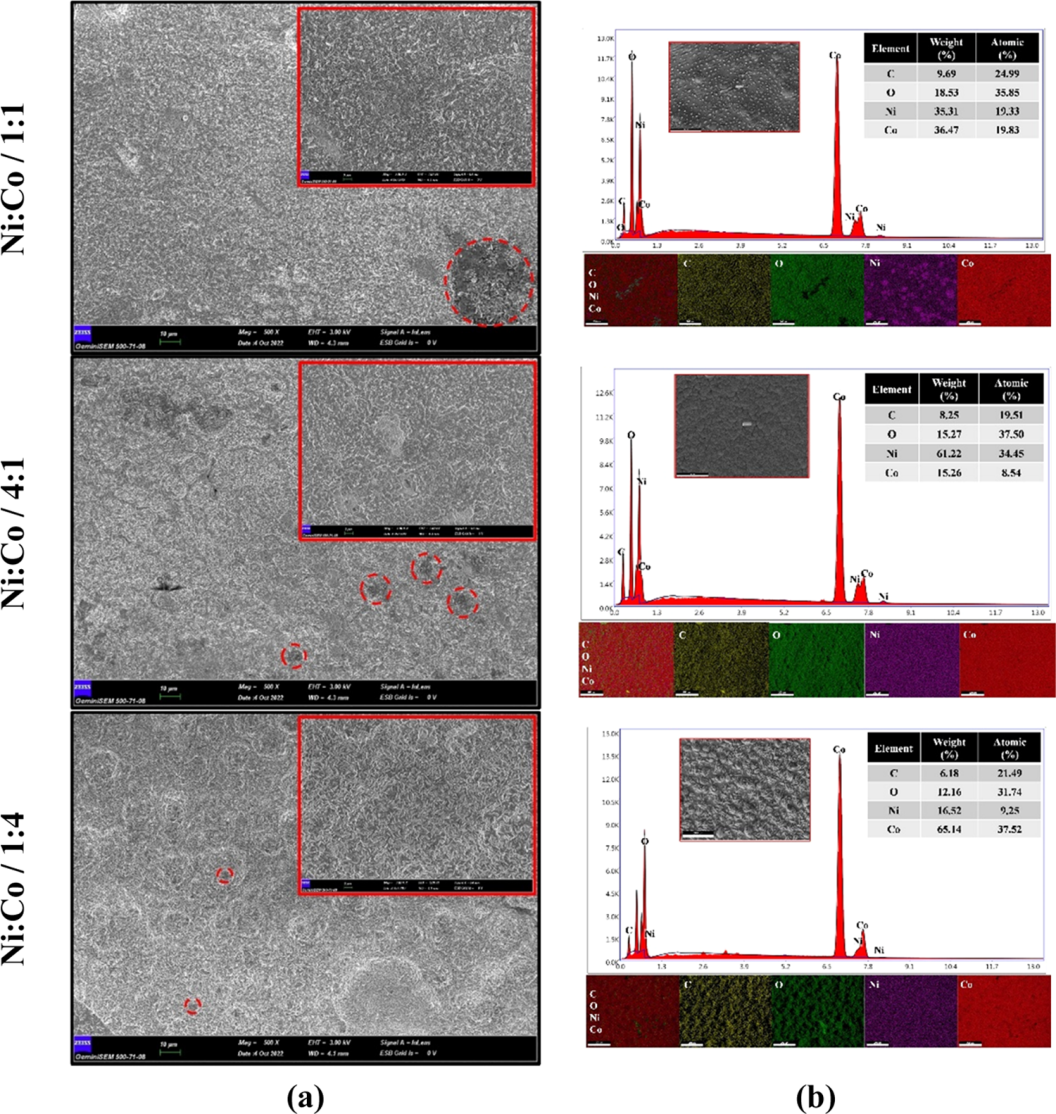
(a) FE-SEM images and (b) FE-SEM/EDX results after CA measurements for 3D electrode samples.

As shown in Figure 11, in the current density of the NiCo-coated electrodes, any noticeable change has not been observed over 72,000 s. The values of current densities for the Ni/Co/1:1-, Ni/Co/4:1-, and Ni/Co/1:4-coated 3D electrodes are measured as 5.27, 8.03, and 12.15 mA/cm^2^, respectively. The measured current density values show that the Ni/Co/1:4-coated 3D electrode is 2.3 times more stable than the Ni/Co/1:1-coated 3D electrode. These results confirmed that the Ni/Co/4:1-coated 3D electrode has high stability due to the durability of current density for HER in a 1 M KOH solution. Moreover, the stability analysis shows that the Ni/Co/1:4-coated electrode is the most stable compared to other coated electrodes and this electrode may maintain its kinetic activity in the electrolysis of water for long-term operations. To further prove the superiority of the stability of the electrodes, we examined the morphology and elemental compositions of the NiCo-coated electrodes via XRD, FE-SEM, and FE-SEM/EDX after CA tests. Figure 12 shows FE-SEM images and FE-SEM/EDX results of NiCo-coated electrodes after CA tests.

**Figure 13:**
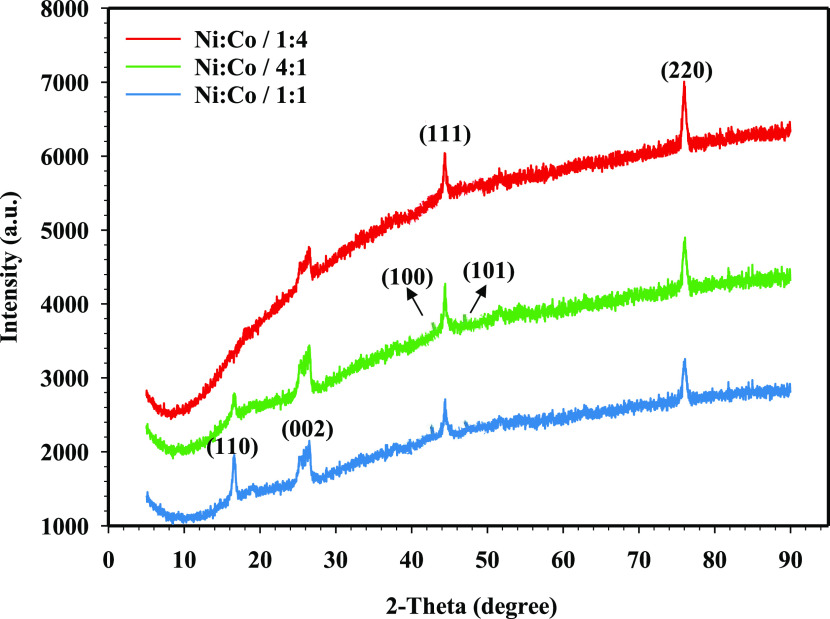
XRD patterns after CA measurements for 3D electrode samples in 1 M KOH.

As seen in Figure 12a, even after the CA for 20 h in an alkaline media, it is observed that NiCo coatings are still present on the 3D electrode surfaces and the particles’ abundance can be observed clearly. It is found that NiCo alloys are dispersed on the electrode surface with granular structures, while specific areas are corroded. Among all coated electrode samples, the Ni/Co/1:1-coated 3D electrode has larger corrosion regions. However, it is determined that the corrosion zones decreased significantly in higher Ni and Co content samples, and the corrosion regions became smaller. According to the FE-SEM/EDX analysis after CA tests, it is determined that there is a carbon peak due to the corrosion on the surface of the NiCo-coated 3D electrodes. As can be seen in Figure 12b, it is found that the carbon peak in the Ni/Co/1:1-coated electrode is 9.69% by weight. On the other hand, this value is less than other Ni- and Co-coated electrodes, and they are obtained as 8.25 and 6.18 wt % for Ni/Co/4:1- and Ni/Co/1:4-coated 3D electrodes, respectively. Moreover, according to the FE-SEM mapping results on the surface of the electrodes, it is seen that the NiCo coatings have a uniform distribution. XRD patterns of the NiCo-coated 3D electrodes after CA for 20 h in 1 M KOH can be seen in Figure 13.

As can be seen in Figure 13, for all of the electrode specimens, two strong peaks are observed for Ni–Co alloys at 44.49 and 76.21°, which are attributed to (111) and (220), respectively. On the other hand, the peaks at 16.525 and 26.53° correspond to the carbon peaks that occurred because of corrosion regions appearing on the electrode surface. It is observed that with the increase of the molar ratios of Ni and Co, the peaks of carbon have gradually decreased on the electrode surface. The intensity of change in angle (2θ(deg)) can be considered as a measure of the stability of the electrodes. This change has occurred as time increased for all electrode samples due to the dissolution of NiCo coatings from the electrode surface. In the XRD analysis performed after the CA test of the electrodes, with the increasing Co content in the coating bath solution, the change in the intensity of the angles gradually decreases. Therefore, it has been indicated that the Ni/Co/1:4-coated electrode has less change than other coated electrodes and exhibits long-term stability.^81^ Moreover, the half-height peak width of the (111) plane of the Ni/Co/1:4-coated electrode at 2θ = 44.49° is determined as β = 0.313, and the size of the (111) particle size has been computed as 28.1 nm. On the other hand, after CA measurements of Ni/Co/1:1- and Ni/Co/4:1-coated electrodes, the grain sizes are found as 25.9 and 26.8 nm, respectively. In addition, it is concluded that the carbon peaks and grain size on the surface of the NiCo-coated electrodes are decreased in the XRD patterns. Considering all of these results, the Ni/Co/1:4-coated 3D electrode has exhibited outstanding stability performance.

## 4 Conclusions

Ni/Co/1:1, Ni/Co/4:1, and Ni/Co/1:4 coatings are coated on a 3D electrode by the electrochemical deposition method successfully. According to the results of FE-SEM, it is seen that the particle size changes in the surface morphology of the NiCo-coated 3D electrodes. Moreover, as seen in the XRD patterns, the particle size of the Ni/Co/1:4-coated 3D electrode has increased by 5.44% with the increase of the Co content. Moreover, XRD analysis proves that the peaks obtained in the structural analysis of the 3D electrodes coated with Ni and Co are NiCo alloys. As a result of LSV measurements, the Ni/Co/1:4-coated 3D electrode has a 5.51 times higher current density value than other electrodes at a −1 V constant voltage. In the CV results, the kinetic activity of the Ni/Co/1:4-coated 3D electrode has enhanced compared to that of the other electrodes, and the current density at 1.1 V is measured as 30.22 mA/cm^2^. The EIS results show that the increase in HER high kinetic activity observed for the Ni/Co/1:4-coated 3D electrode is due to the decreased charge-transfer resistance. It is observed that the *R*_ct_ value of NiCo-coated 3D electrode samples has decreased from 0.340 to 0.306 depending on the ratio of Ni and Co. Also, the lowest resistance and CPE values have occurred in the Ni/Co/1:4-coated 3D electrode, which has the best kinetic activity for the HER in an alkaline medium. According to the results, it is concluded that the Ni/Co/1:4-coated 3D electrode shows higher kinetic activity and stability than the other 3D electrodes. The combination of a large kinetic active surface area because of Co addition, the interaction between Ni–Co, and the formation of Ni and Co on the 3D electrode surface results in good properties for HER application. Moreover, it is presented that 3D electrodes, which are prepared along with their fast and low cost, have better kinetic activity and stability for HER, and the electrodes can be used as cathode materials for alkaline electrolyzers. Therefore, this study provides great potential in improving high-performance 3D electrodes for many different electrochemical energy conversions and storage applications, such as electrolysis, supercapacitors, and batteries. In our future studies, we investigate the effect of different electrode geometries with noble metal coatings in alkaline and other types of electrolyzers.

## Supporting Information Available

Details of the elemental properties and surface characterization of NiCo-coated 3D electrodes (PDF)
