# Supplementary material for: Hydrogen Evolution Reaction Performance of Ni–Co-Coated Graphene-Based 3D Printed Electrodes
Source: ACS Omega. 2023 Feb 2;8(6):5958–74. doi: 10.1021/acsomega.2c07856 (PMC9933213; doi:10.1021/acsomega.2c07856)
Supplement: Supplementary file 1 — ao2c07856_si_001.pdf [file ao2c07856_si_001.pdf]

Supporting Information

Hydrogen Evolution Reaction Performance of Ni-Co Coated Graphene-Based 3D Printed Electrodes

Bulut Hüner [1,2,3], Nesrin Demir [1,3*], Mehmet Fatih Kaya [1,3,4]

[1]Erciyes University, Engineering Faculty, Energy Systems Engineering Department, Heat Engineering Division, 38039, Kayseri, Turkey

[2]Erciyes University, Graduate School of Natural and Applied Sciences, Energy Systems Engineering Department, 38039, Kayseri, Turkey

[3]Erciyes University H2FC Hydrogen Energy Research Group, 38039, Kayseri, Turkey

[4]BATARYASAN Enerji ve San.Tic. Ltd.Şti, Yıldırım Beyazıt Mah., Aşık Veysel Bul., ERÜ TGB Kuluçka Merkezi, No: 63/B, 38039 Kayseri, Turkey

* Corresponding author. Tel.: +90 3522076666 / 32330.

E-mail address: nkayatas@erciyes.edu.tr

(a)

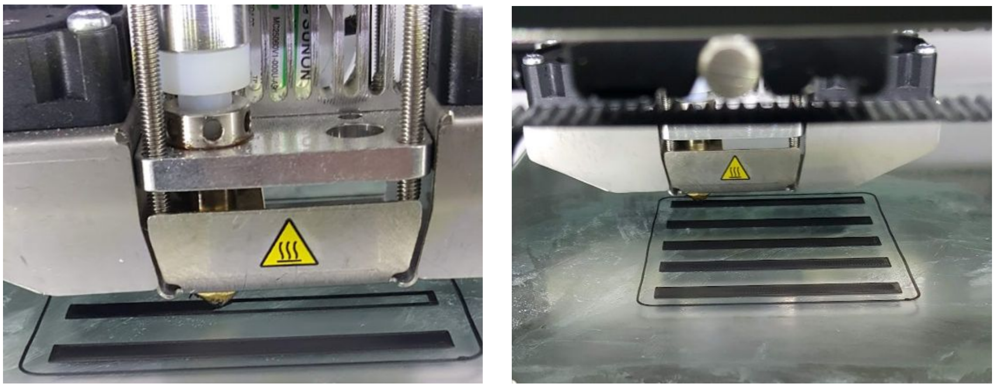

(b)

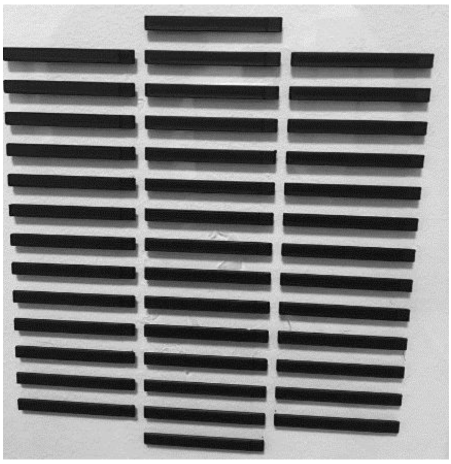

Figure S1. (a) 3D printing process of the electrode samples and (b) completed 3D printed electrode samples.

(a)

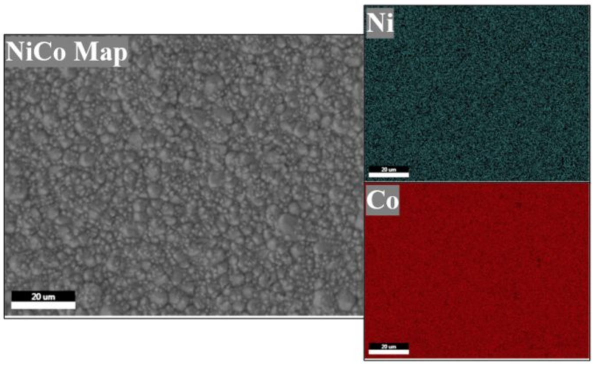

(b)

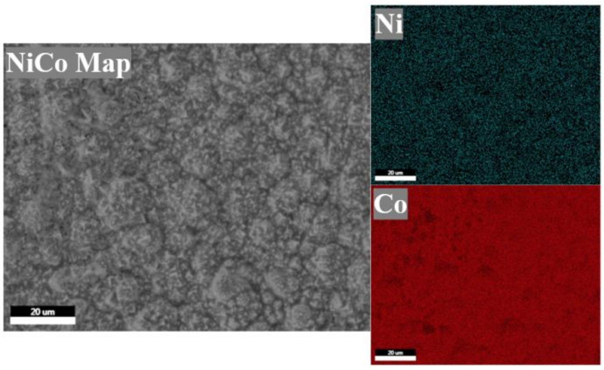

(c)

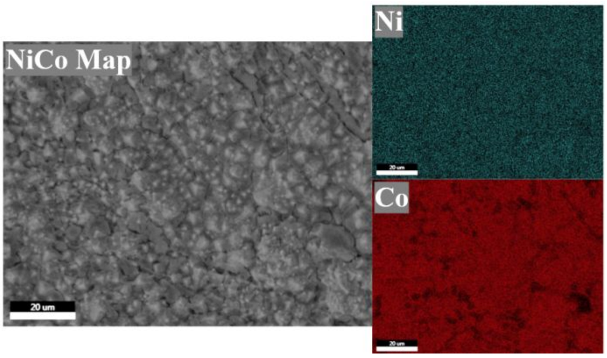

Figure S2. FE-SEM image mapping of NiCo coated 3D electrode samples a) Ni:Co / 1:1, b) Ni:Co / 4:1, and c) Ni:Co / 1:4.

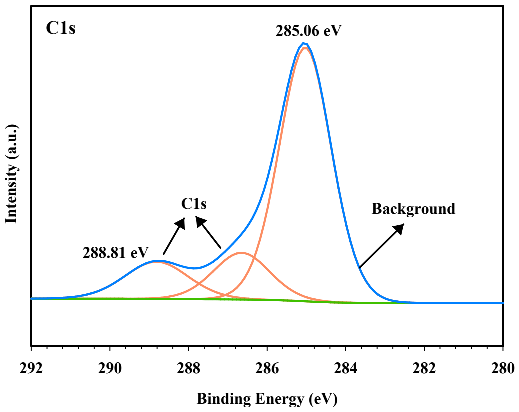

(a)

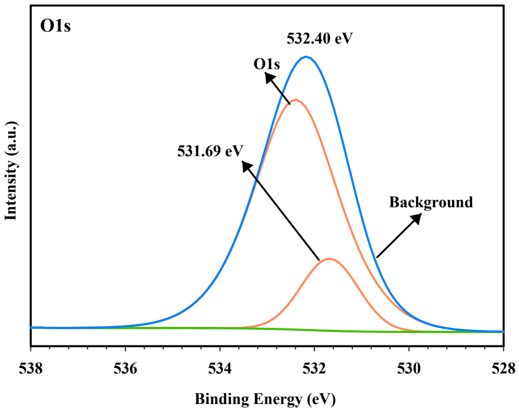

(b)

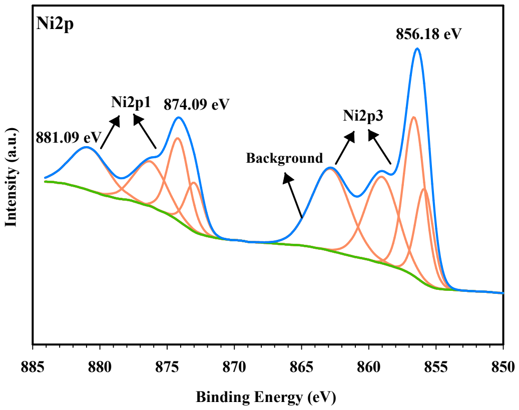

(c)

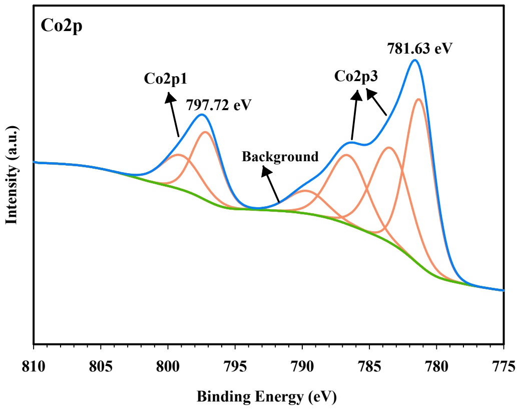

(d)

Figure S3. XPS spectra of Ni:Co / 1:1 electrode; (a) C1s, (b) O1s, (c) Ni2p and (d) Co2p.

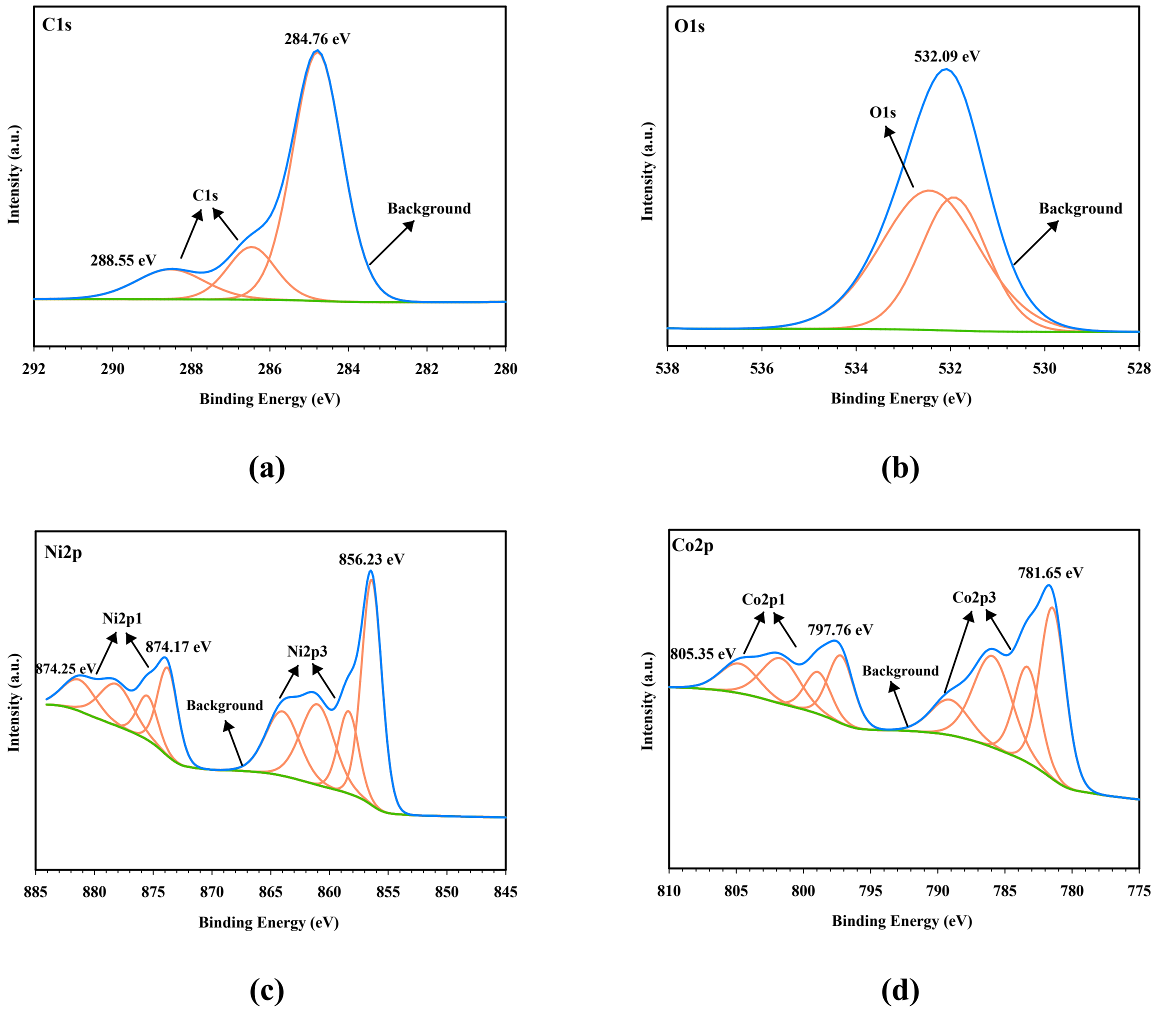

Figure S4. XPS spectra of Ni:Co / 4:1 electrode; (a) C1s, (b) O1s, (c) Ni2p and (d) Co2p.

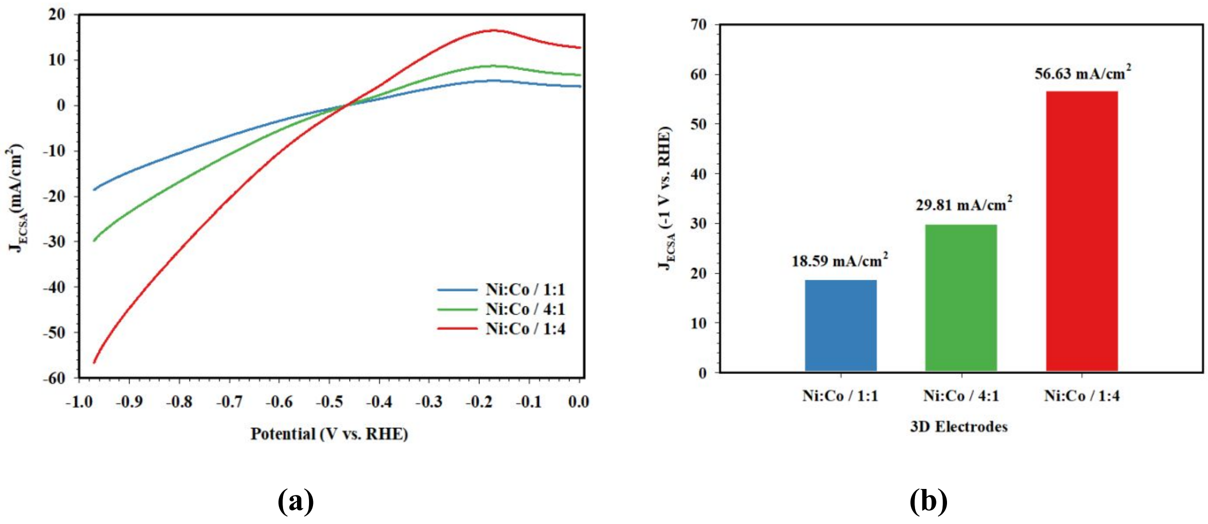

Figure S5. (a) ECSA normalized HER polarization curve of NiCo coated 3D electrodes, and (b) Corresponding J_{ECSA} at -1 V vs. RHE (J_{ECSA}: current density normalized by ECSA).
